# The prevalence of occult hepatitis B virus (hbv) infection in a large multi-ethnic haemodialysis cohort

**DOI:** 10.1186/s12882-015-0010-z

**Published:** 2015-02-06

**Authors:** Luciana Sowole, Wendy Labbett, Mauli Patel, Aisling O’Riordan, Jennifer Cross, Andrew Davenport, Tanzina Haque

**Affiliations:** Department of Virology, Royal Free Hospital, Pond Street, Hampstead, London, NW3 2QG UK; Centre for Nephrology, Royal Free Hospital, Pond Street, Hampstead, London, NW3 2QG UK; UCL Centre for Nephrology, Royal Free Hospital, University College London Medical School, Rowland Hill Street, Hampstead, London, NW3 2PF UK

**Keywords:** Haemodialysis, Hepatitis B virus, Occult infection

## Abstract

**Background:**

Haemodialysis patients are at increased risk of exposure to blood borne viruses. To reduce transmission in the UK, all haemodialysis patients are regularly screened, and if susceptible to Hepatitis B virus (HBV) infection, vaccinated.

**Methods:**

This retrospective study was undertaken to determine the HBV immune status in a large dialysis cohort and the prevalence of occult HBV infection, defined as the presence of anti-HBcore antibody (anti-HBcAb) and HBV DNA without detectable HB surface antigen (HBsAg). Information on HBV status was retrieved from haemodialysis patients under the care of The Royal Free Hospital, London, UK between 2009–2010. Available sera from 138 of 161 anti-HBcAb positive/HBsAg negative individuals were anonymised and tested for HBV DNA by a real time quantitative PCR.

**Results:**

15 (2%) of 793 patients had chronic HBV infection (HBsAg positive). 161 (20%) were anti-HBcAb positive but HBsAg negative suggesting past infection. 335 (54%) of the remaining 617 patients were considered immune following vaccination (anti-HBsAb > 10 IU/L). Three (2.2%) of the 138 anti-HBcAb positive, HBsAg negative patients had detectable HBV DNA (3, 5 and 9 IU/ml). Standard liver function tests were normal in these patients.

**Conclusions:**

In a large multi-ethnic London haemodialysis cohort, 20% patients had evidence of past HBV infection. Despite this, the prevalence of occult HBV was found to be low and the very low levels of HBV DNA detected are unlikely to pose a nosocomial transmission risk in the presence of robust vaccination and infection control measures.

## Background

Haemodialysis patients are at increased risk of infections with blood-borne viruses (BBV), such as hepatitis B virus (HBV), hepatitis C virus (HCV) and human immunodeficiency virus (HIV). Current UK guidelines recommend that patients on haemodialysis are routinely tested for BBV and that all dialysis centres implement measures to prevent nosocomial transmission.

Many new infections with HBV are sub-clinical, and current infection can be detected by the presence of Hepatitis B surface antigen (HBsAg) in the serum [1]. Following the discovery that patients infected with HBV could transmit infection within a haemodialysis unit, a code of practice was introduced into the UK in the early 1970s, which dramatically reduced the incidence of HBV infections in UK dialysis patients and staff [2]. UK government Department of Health (DOH) guidance recommends that haemodialysis patients are screened 3 monthly for HBsAg, and chronic HBV patients are dialysed in isolation using dedicated machines with strict infection control measures to prevent nosocomial transmission. Those patients returning from ‘dialysis away from base’ in resource poor countries undergo enhanced screening for HBsAg for 8 weeks [3].

The presence of HBV core antibody (anti-HBcAb) in blood with or without anti-HB surface antibody (anti-HBsAb) is considered as evidence of past HBV infection, and these patients are considered non-infectious and HBV DNA is therefore not routinely tested in this group. However, occult HBV infection, defined as the presence of anti-HBcAb and HBV DNA in blood without any detectable HBsAg, has been described in these ‘HBV past infection’ patients. As such, it has been suggested that there may be a potential risk of patients with occult HBV transmitting infection within dialysis units, as these patients are not isolated or segregated from other dialysis patients.

One of the mainstays of preventing HBV transmission within haemodialysis units is the establishment of a robust vaccination programme. Patients with chronic kidney disease (CKD) exhibit specific and non-specific defects in both humoral and cellular immune responses [4]. As a result, the response to Hepatitis B vaccination is lower in haemodialysis patients compared with the general population. Vaccination is therefore advised early in the course of the renal disease, using a double vaccine dose (40 microgrammes) and a 4 rather than 3 dose schedule [5]. It is estimated that 45–66% of patients with CKD develop adequate anti-HBs responses, and however levels decline more rapidly in comparison with immuno-competent individuals [6]. Anti-HBsAb levels above 10 IU/L are considered to be protective.

### Objectives

We wished to determine the HBV immune status of our cohort and the prevalence of occult HBV infection in a large inner city haemodialysis program following current UK guidelines for HBV vaccination, and whether occult HBV infection posed a potential risk for transmission within a haemodialysis unit despite following national policy.

## Study design and methods

The study population comprised 793 adult patients undergoing haemodialysis at The Royal Free Hospital and its satellite dialysis units in 2009–2010. Demographic data on age and ethnic origin were collected from the Renal database. A systematic search for Virology results (using the Pathology reporting system) was carried out for each patient. Information was gathered on the Hepatitis B surface antigen (HBsAg), core total antibody (anti-HBc), surface antibody (anti-HBs), Hepatitis C and HIV status. Note was made of the date of administration of each vaccination, and if no such record existed, it was recorded as ‘no vaccination documented’. The data was then analysed by initially identifying patients with current or past Hepatitis B infection and categorising the remaining patients according to their immune status i.e. those with an anti-HBs level > 10 IU/L were classified as immune versus <10 IU/L (non-immune). Stored serum samples from anti-HBcAb positive, HBsAg negative patients were anonymised and tested for HBV DNA by an in-house real time quantitative PCR assay with a lower level of detection of 1.5 IU/ml [7].

Statistical analysis was performed using Chi Square test with Yates Correction, using GraphPad Prism 5 Graph Pad, San Diego, USA). HBV immunity was compared between the White ethnic group and other ethnic groups (excluding the ‘not stated’ groups).

This retrospective audit complied with the UK National Health Service (NHS) guidelines for clinical audit and service development, and had appropriate approval. Individual patient consent was waived by the Royal Free Hospital Research and Development office as the audit complied with NHS guidelines (UK NHS guidelines for clinical audit and service development, available at http://www.hra-decisiontools.org.uk/ethics/, and http://www.hra.nhs.uk/research-community/before-you-apply/determine-whether-your-study-is-research/). Anonymised data were collected from virology laboratory tests that had been performed as part of the routine clinical care of kidney dialysis patients in keeping with the Royal Free Hospital Trust policy and no patient identifiable data was used. No children were included in this audit of adult patients.

## Results

**Figure 1:**
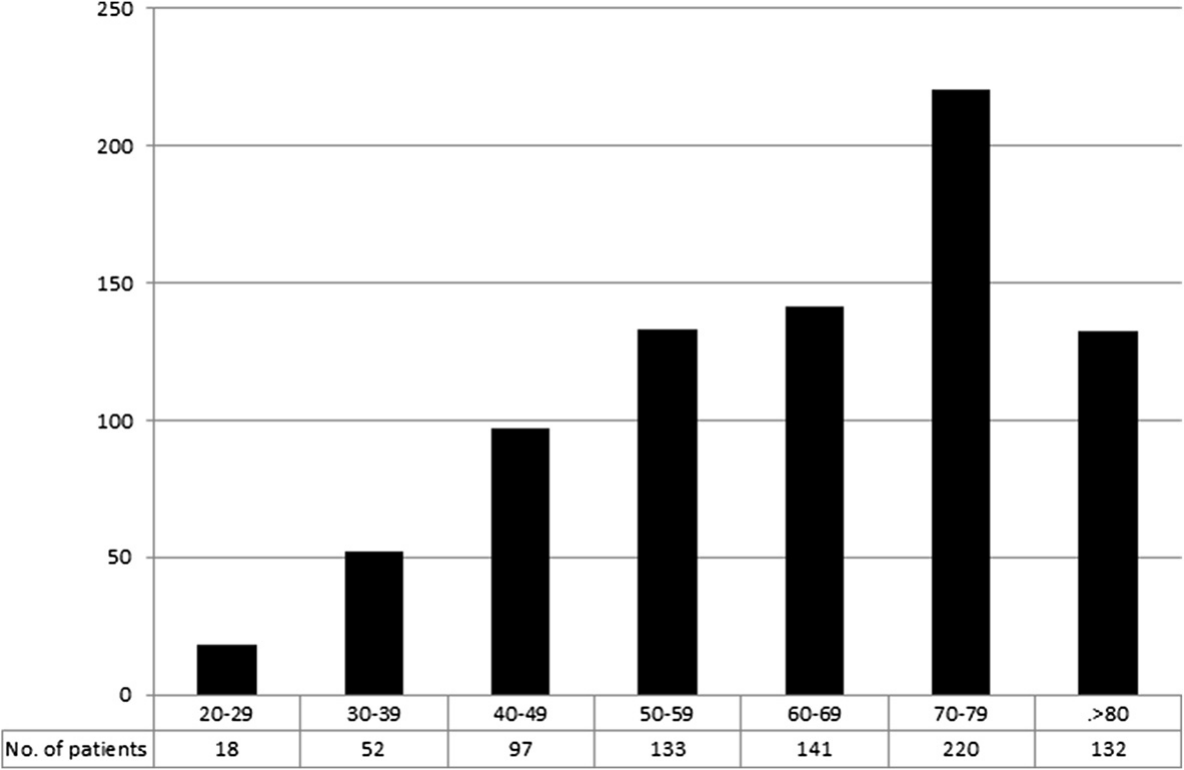
**Distribution of patient age.**

**Figure 2:**
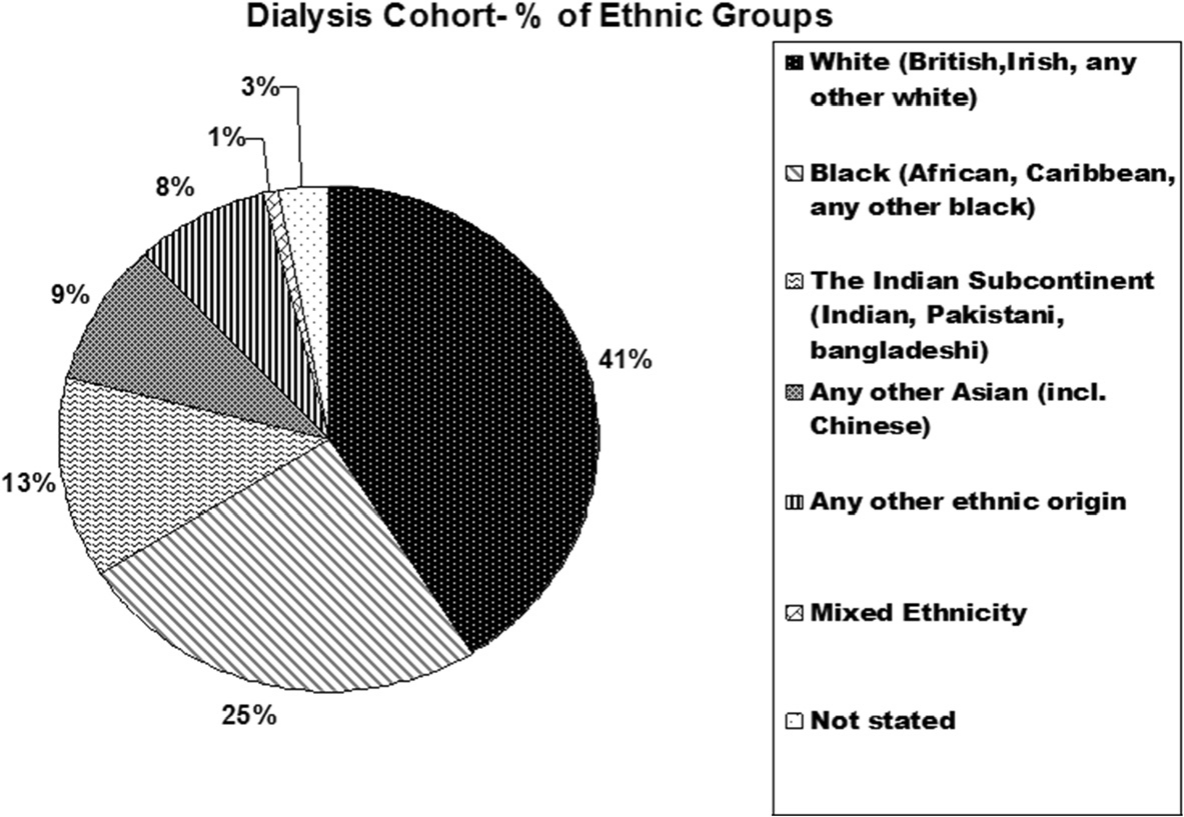
**Distribution of patient ethnicity.**

The demographic characteristics of the dialysis population are shown in Figures 1 and 2. Age ranged from 23 to 99 years with a median of 66 years. Seventy nine percent of the patients were ≥ 50 years of age (Figure 1 distribution of patient ages). This inner city patient cohort came from a wide range of ethnic backgrounds (Figure 2 distribution of patient ethnicity).

Fifteen (2%) of the 793 patients were HBsAg positive and anti-HBcAb positive for over at least 6 months or more with detectable HBV DNA in blood indicating chronic HBV infection. A total of 161 patients (20%) were anti-HBcAb positive but HBsAg negative and were therefore considered to have had past HBV infection (Table 1). One hundred and thirty nine patients (86%) also had HBsAb and the remaining 22 (14%) were isolated HBcAb positive. Of note, patients with past HBV infection were more likely to be from ethnic groups other than White (79% other ethnic groups versus 18% White (Table 2 distribution of patient ethnicity).

**Table 1:** **Hepatitis B status of our haemodialysis cohort**

	**Hepatitis B surface anigen (HBsAg)**	**Hepatitis B core antibody (anti-HBcAb)**	**Hepatitis B surface antibody (anti-HBsAb)**	**Number of patients**
**HBV immune via vaccination**	Negative	Negative	Positive	335 (42%)
**Chronic HBV infection**	Positive	Positive	Negative	15 (2%)
**Past Hepatitis B infection**	Negative	Positive	Positive	139 (17%)
Past Hepatitis B infection*	Negative	Positive	Negative	22 (3%)
**Non-immune**	Negative	Negative	Negative	282 (36%)

*Patients who have acquired natural immunity due to prior exposure to hepatitis B.

**Table 2:** **‘Past’ versus ‘Vaccine-induced’ immunity in the different ethnic groups of the dialysis cohort**

**Ethnicity**	**Past hepatitis B infection**	**Vaccine induced immunity**
**White (British, Irish, any other white)**	29	147
**Black (African, Caribbean, any other black)**	73	77
**The Indian Subcontinent (Indian, Pakistani, Bangladeshi)**	19	49
**Any other Asian (incl. Chinese)**	16	25
**Any other ethnic origin**	15	26
**Mixed Ethnicity**	4	4
**Not stated**	5	7
TOTAL NO.	161	335

335 of the remaining 617 patients (54%; overall 42% of 793 patients) were immune to HBV through vaccination with anti-HBsAb levels of >10 IU/L (Table 1). 282 patients (46%) were non-immune to HBV. The ethnicities of those with vaccine induced immunity are presented in Table 2. Analysis of our data showed that patients from the Caucasoid ethnic group were more likely to respond to vaccination and developed immunity compared with patients from the other ethnic groups (two-tailed p = 0.041).

Serum samples were available for HBV DNA testing from 138 of the 161 patients who were anti-HBcAb positive and HBsAg negative. Of these, 126 patients also had anti-HBsAb. Three of 138 anti-HBcAb positive patients had very low levels of HBV DNA detected by PCR (3, 5 and 9 IU/ml respectively). The prevalence of occult HBV infection was therefore 2.2% in the test group, and 0.4% in the overall dialysis cohort. One patient was anti-HBcAb positive/anti-HBs Ab positive (from ‘any other ethnic group’) and two were isolated anti-HBc Ab positive (from ‘any other ethnic group- Greek’ and ‘other Asian origin’- Chinese). Standard liver function tests were within the normal reference ranges in all three of these patients, and no patient had a history of liver disease, or co-infection with hepatitis C or blood transfusion prior to the positive sample.

Twenty five (3%) out of the 793 patients had current hepatitis C infection and were hepatitis C RNA positive. The prevalence of HIV antibody positivity (with or without HIV RNA suppression) in our cohort was 2% (16 out of 793).

## Discussion

Occult Hepatitis B infection is characterized by the presence of HBV DNA without detectable HBsAg, with or without the presence of HBV antibodies outside the acute phase window period [8]. A number of possible mechanisms have been suggested for the pathogenesis of occult Hepatitis B infection, although it is most likely multifactorial, depending upon both host and viral factors. The majority of cases are secondary to overt HBV infection and represent a residual low level viraemia suppressed by a robust immune response, together with abnormal histological findings on liver biopsy which developed either during the acute or chronic phase of HBV infection [9,10].

In our study, a sensitive real time quantitative PCR assay was used to determine the presence of occult Hepatitis B infection in a large cohort of inner city adult patients receiving maintenance haemodialysis. We did find occult HBV, although the prevalence and levels of detectable circulating HBV were low.

Some studies have observed a low level of occult HBV in haemodialysis patients in countries that may not employ the isolation and screening policies in combination with active vaccination followed in the UK. In a multicentre study of 289 haemodialysis patients in Tehran, Iran, Aghakhani et al. reported that HBV DNA was detected at levels <50 IU/ml in 9 of the 18 patients who were anti-HBcAb positive/anti-HBsAb negative [11]. On the other hand, Fabrizi et al. isolated anti-HBcAb in 20.8% of their Italian patients but did not find any cases of occult HBV in their study group [12]. Similarly Jardim et al. also did not detect HBV DNA in 34 haemodialysis patients dialysing in Brazil who were anti-HBcAb positive [13].

In non-haemodialysis patients Kang et al., described 4 out of 230 Korean patients with ‘anti-HBcAb alone’ were found to have very low levels of HBV DNA [14]. Vitale et al. from Italy, set out to determine whether anti-HBcAb in isolation can be used as a marker of ‘occult’ HBV in patients with or without HCV infection. The patient group consisted of asymptomatic outpatients referred for routine testing for viral hepatitis, drug users and patients with hepatocellular carcinoma (HCC). A total of 223 sera from non-haemodialysis patients were found to have ‘anti-HBcAb alone’, and of these 9 (4.0%) patients were found to have a detectable HBV DNA [15].

The prevalence of HBV infection varies from country to country, and as such one would expect that occult HBV would similarly vary with geographic distribution. Previous smaller studies have supported our findings that overall rates of occult HBV in haemodialysis cohorts appear to be low. In the vast majority of these occult ‘infections’ the viral load levels are also consistently low. Although in theory these patients may pose a nosocomial transmission risk, the detection of HBV DNA does not always indicate infectivity or disease progression, therefore it has been proposed that a more comprehensive term such as ‘occult Hepatitis B’ rather than ‘occult Hepatitis B infection’ is used [16].

It is difficult to ascertain the exact levels of HBV DNA in blood that may lead to transmission in the haemodialysis setting. The UK Department of Health guidelines recommend that HBV infected healthcare workers can be allowed to perform exposure prone procedures if their HBV DNA level is suppressed to <1,000 copies/ml (around 250 IU/ml) [17].

Although we follow the current UK practice of isolating patients with chronic hepatitis B infection, and implement practices designed to prevent nosocomial infection within our dialysis centres, we did detect a number of patients with occult hepatitis B which may reflect our inner city, multi-ethnic practice, as in our haemodialysis cohort, 62.5% of patients are considered HBV immune, either through vaccination of due to past infection. Although we could detect circulating HBV in a small number of HBsAg negative patients, the low levels of circulating HBV DNA we detected in our cohort are unlikely to pose a real risk of nosocomial transmission in clinical practice based on following universal precautions, and particularly if a robust HBV vaccination programme is implemented in haemodialysis units. However it is especially important that vaccination is provided early in patients with CKD thought to be at risk of requiring renal replacement therapy, as it has been shown that these individuals have an impaired response to vaccination once established on haemodialysis. In our cohort, patients from the Caucasoid ethnic group showed better vaccination-induced immune response than patients from other ethnic groups (p = 0.041). The ethnic difference in vaccine response is not well understood, although a recent study suggests that certain human leukocyte antigen (HLA) tissue types are associated with non-responsiveness to HBV vaccination and that different HLA types of the ethnic groups should be considered when evaluating vaccine responses [18].

## Conclusions

The strength of our audit is our large multi-ethnic haemodialysis cohort in North Central London, with 20% of patients having evidence of previous infection with HBV. Despite this, the prevalence of patients having circulating detectable HBV viraemia (occult HBV) was found to be low and the reassuringly the very low levels of HBV DNA detected are very unlikely to pose a nosocomial transmission risk to other kidney haemodialysis patients in the presence of robust active vaccination program against HBV and appropriate infection control measures. As we only measured HBV on one occasion the limitation of our study is that we cannot exclude that at other times patients may have higher circulating viral DNA levels and may pose a risk of blood borne infection which could be increased in centres without an active vaccination program or infection control policies.
